# Identification and verification of the molecular mechanisms and prognostic values of the cadherin gene family in gastric cancer

**DOI:** 10.1038/s41598-021-03086-1

**Published:** 2021-12-08

**Authors:** Shanshan Luo, Rujing Lin, Xiwen Liao, Daimou Li, Yuzhou Qin

**Affiliations:** 1 Department of Gastrointestinal Surgery, Guangxi Medical University Cancer Hospital, Guangxi Clinical Research Center for Colorectal Cancer, He Di Road 71, Nanning, 530021 Guangxi Autonomous Region People’s Republic of China; 2 Department of General Surgery, The People’s Hospital of Binyang County, Nanning, 530405 Guangxi Zhuang Autonomous Region People’s Republic of China; 3 Department of Hepatobiliary Surgery, The First Affiliated Hospital of Guangxi Medical University, Nanning, 530021 Guangxi Zhuang Autonomous Region People’s Republic of China

**Keywords:** Cancer, Genetics, Biomarkers, Gastroenterology

## Abstract

While cadherin (CDH) genes are aberrantly expressed in cancers, the functions of *CDH* genes in gastric cancer (GC) remain poorly understood. The clinical significance and molecular mechanisms of CDH genes in GC were assessed in this study. Data from a total of 1226 GC patients included in The Cancer Genome Atlas (TCGA) and Kaplan–Meier plotter database were used to independently explore the value of CDH genes in clinical application. The TCGA RNA sequencing dataset was used to explore the molecular mechanisms of CDH genes in GC. Using enrichment analysis tools, CDH genes were found to be related to cell adhesion and calcium ion binding in function. In TCGA cohort, 12 genes were found to be differentially expressed between GC para-carcinoma and tumor tissue. By analyzing GC patients in two independent cohorts, we identified and verified that CDH2, CDH6, CDH7 and CDH10 were significantly associated with a poor GC prognosis. In addition, CDH2 and CDH6 were used to construct a GC risk score signature that can significantly improve the accuracy of predicting the 5-year survival of GC patients. The GSEA approach was used to explore the functional mechanisms of the four prognostic CDH genes and their associated risk scores. It was found that these genes may be involved in multiple classic cancer-related signaling pathways, such as the Wnt and phosphoinositide 3-kinase signaling pathways in GC. In the subsequent CMap analysis, three small molecule compounds (anisomycin, nystatin and bumetanide) that may be the target molecules that determine the risk score in GC, were initially screened. In conclusion, our current study suggests that four CDH genes can be used as potential biomarkers for GC prognosis. In addition, a prognostic signature based on the CDH2 and CDH6 genes was constructed, and their potential functional mechanisms and drug interactions explored.

## Introduction

Like most solid tumors, gastric cancer (GC) is driven by both genomic and environmental factors. Recent advances in genomics techniques and high throughput analysis allow for the high resolution study of GC at a molecular level. This multi-omics, high-throughput sequencing data has greatly facilitated the identification of possible GC-associated variants, which may include gene and chromosomal mutations, as well as transcriptional and epigenetic alterations^1^. Importantly, an understanding of the potential variants or molecular drivers involved in the pathogenesis of GC can lead to the discovery of important clinical biomarkers and potential therapeutic targets. The Cancer Genome Atlas (TCGA) is an example of a complete multi-group, high-throughput sequencing data set representative of 33 cancers. This data are accompanied with complete, open access, prognostic clinical data that affords researchers with multiple analyses and data mining opportunities^2^. GC-associated multi-omics, high-throughput sequencing data is also contained in TCGA^3^. The cadherin (CDH) gene family, which mainly mediates intercellular adhesion and was found by Takeichi et al. is one of the earliest adhesion molecules described^4^. While *CDH* gene adhesion molecules are essential for selective aggregation of cells during growth and development, in cancer they are closely related to tumor cell invasion and metastasis^5^. After a literature search it was found that there is still no comprehensive study on the relationship between CDH mRNA and GC prognosis. This study therefore endeavored to comprehensively explore the potential functional mechanism of *CDH* genes and their prognostic application value in gastric cancer through two independent GC cohorts.

## Results

### Functional enrichment of cadherin genes

**Figure 1:**
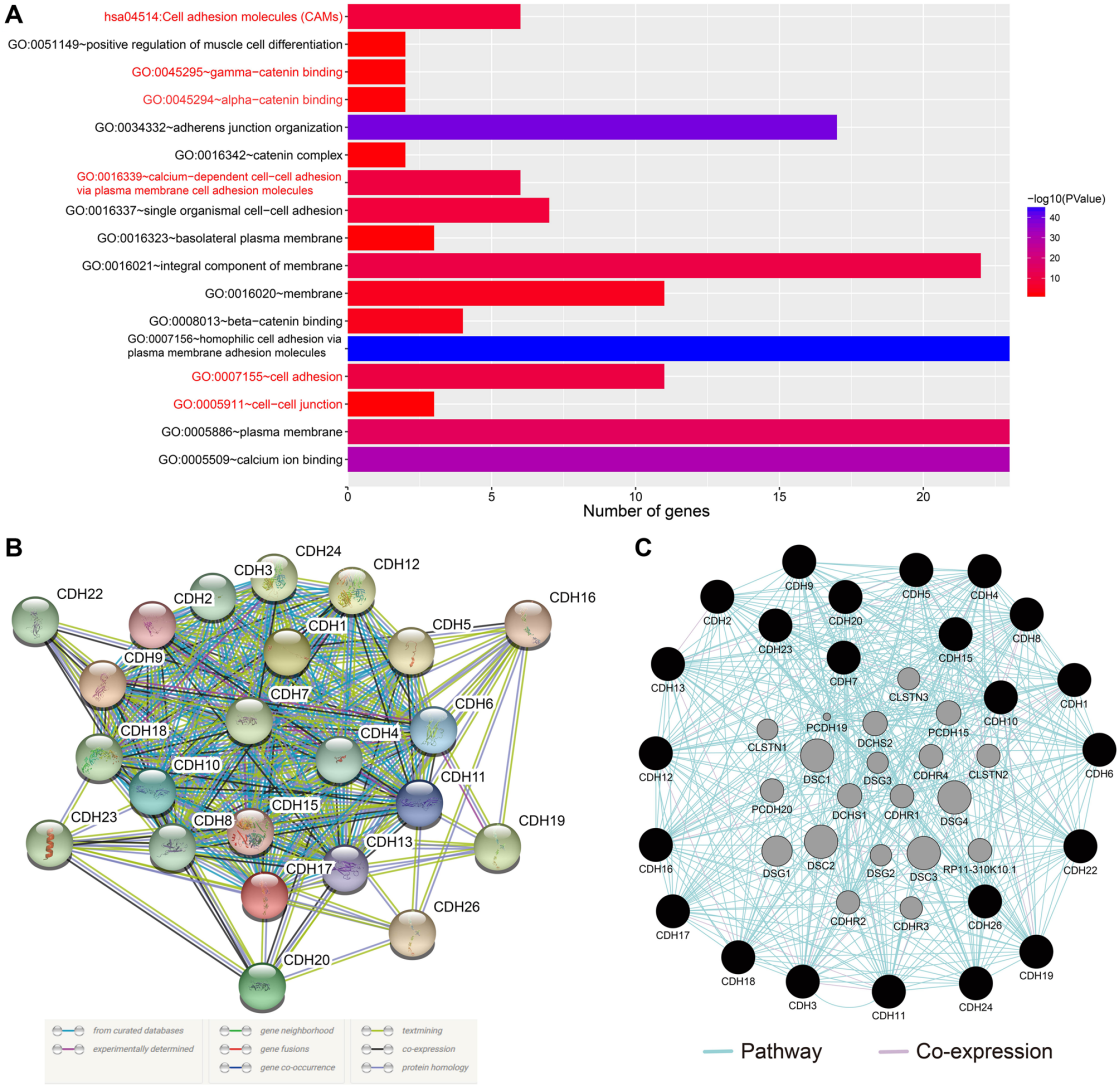
Functional enrichment and gene–gene interaction network analysis of CDH family genes. (**A**) Functional enrichment analysis of CDH family genes; gene–gene interaction network analysis of CDH family genes using STRING (**B**) and GeneMANIA (**C**).

Through functional enrichment analysis, the main functions of cadherin genes were found to include calcium ion binding and participation in various biological processes involving cell adhesion (Fig. 1A).

**Figure 2:**
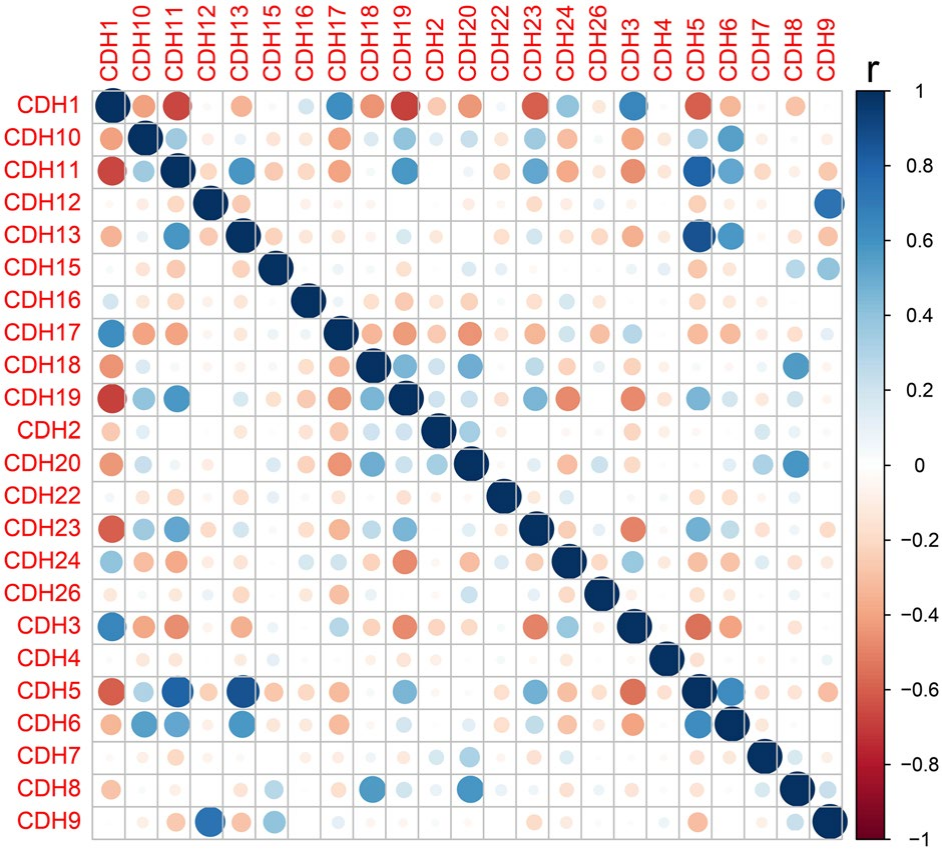
Co-expression matrix heat map of CDH gene family.

Gene–gene interaction analysis revealed significant co-expression and pathway interactions among the CDH genes (Fig. 1B,C). Subsequently, in order to verify the co-expression interaction relationship of these genes in GC tumor tissue, the *cor* function was used to calculate the co-expression correlation coefficient of these genes in R. These CDH genes were also found to have significant co-expression gene–gene interactions in GC tumor tissue (Fig. 2, Table S1).

### Clinical significance of cadherin genes

**Figure 3:**
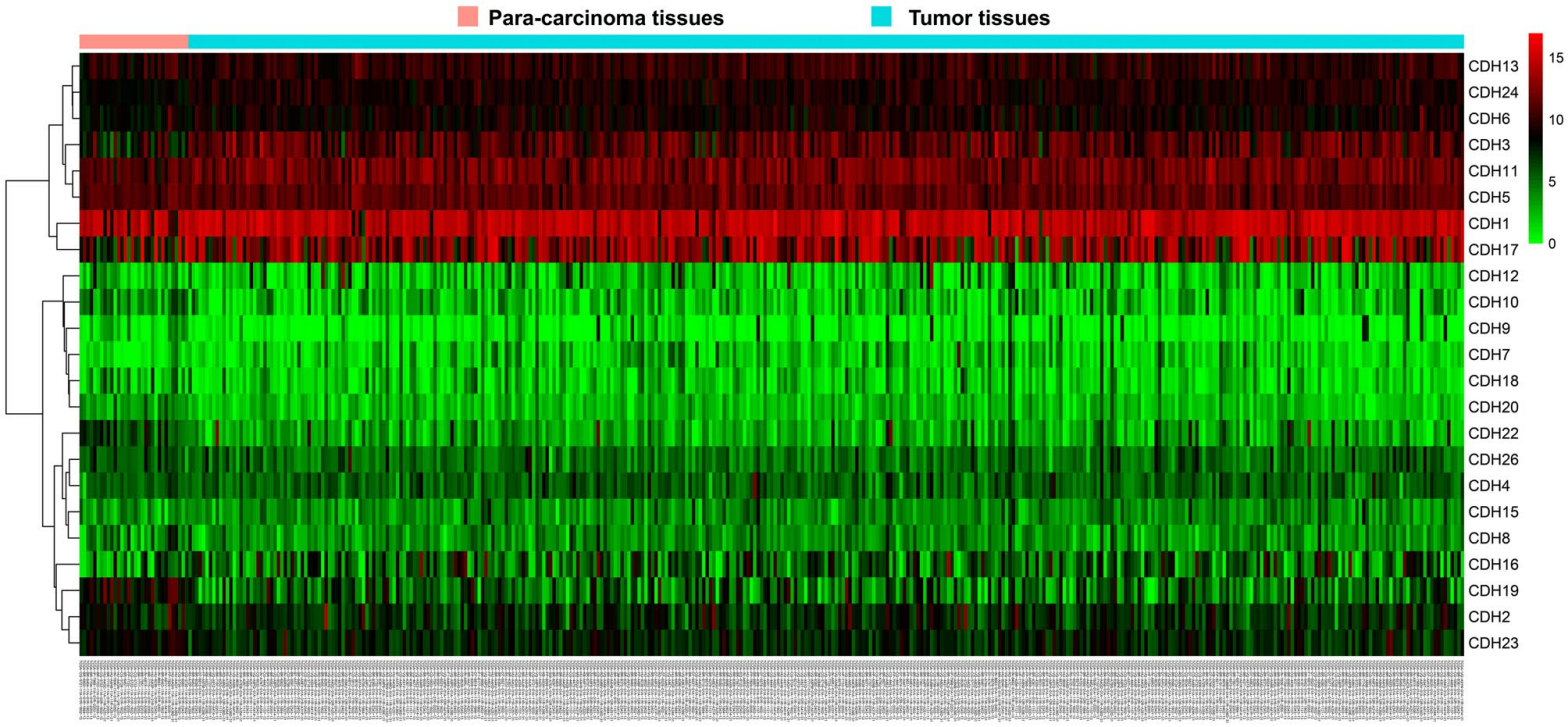
Heat map of CDH family genes expression distribution between GC para-carcinoma and tumor tissues.

**Figure 4:**
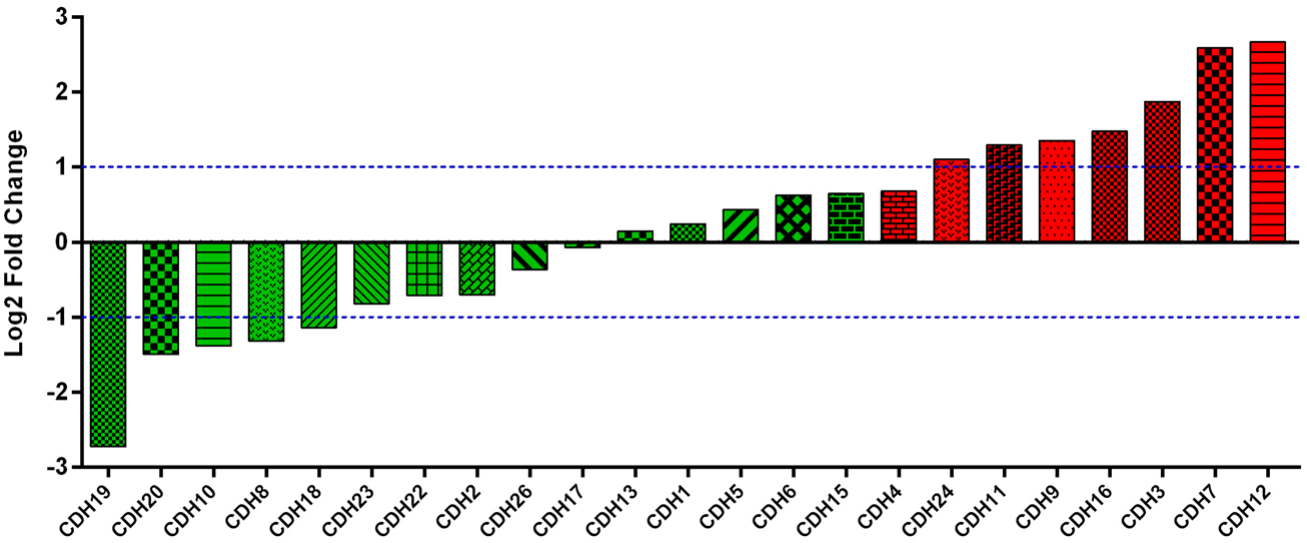
Histogram of the fold change of CDH family genes between GC para-carcinoma and tumor tissues.

**Figure 5:**
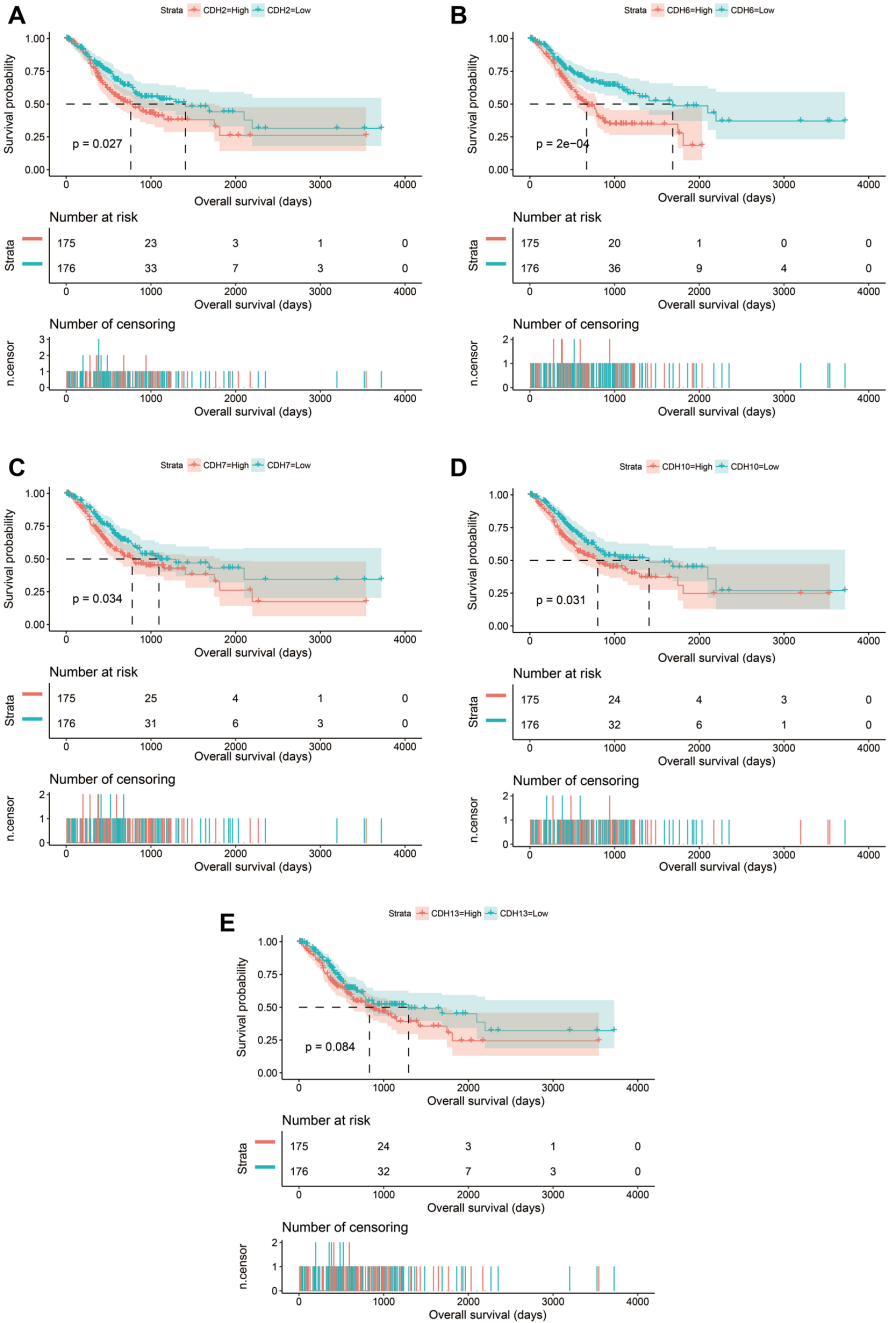
Kaplan–Meier survival curves of prognostic-related CDH genes in TCGA cohort. (**A**) Kaplan–Meier curve of CDH2; (**B**) Kaplan–Meier curve of CDH6; (**C**) Kaplan–Meier curve of CDH7; (**D**) Kaplan–Meier curve of CDH10; (**E**) Kaplan–Meier curve of CDH13.

**Figure 6:**
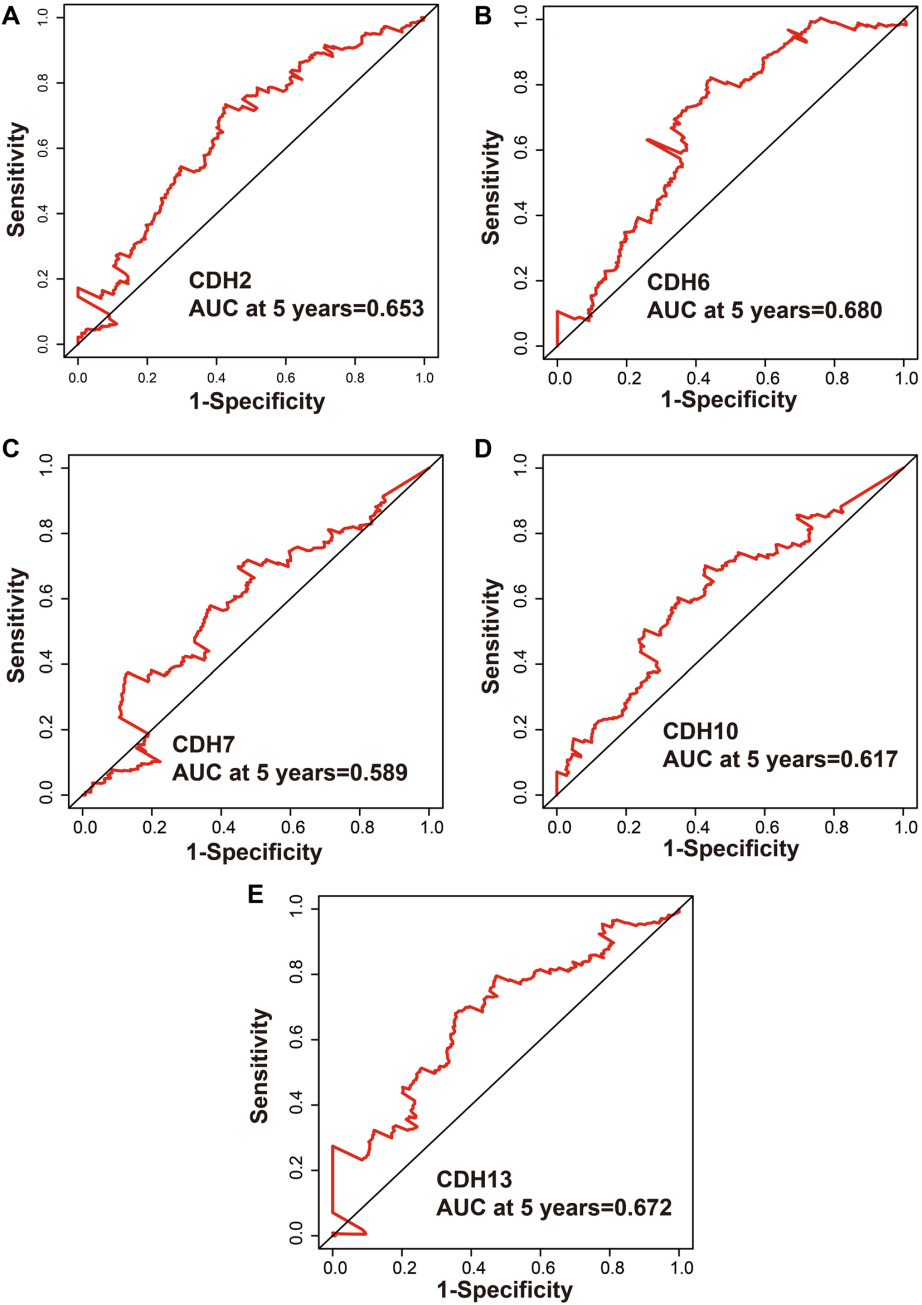
SurvivalROC curves of prognostic-related CDH genes in TCGA cohort. (**A**) ROC curve of CDH2; (**B**) ROC curve of CDH6; (**C**) ROC curve of CDH7; (**D**) ROC curve of CDH10; (**E**) ROC curve of CDH13.

**Figure 7:**
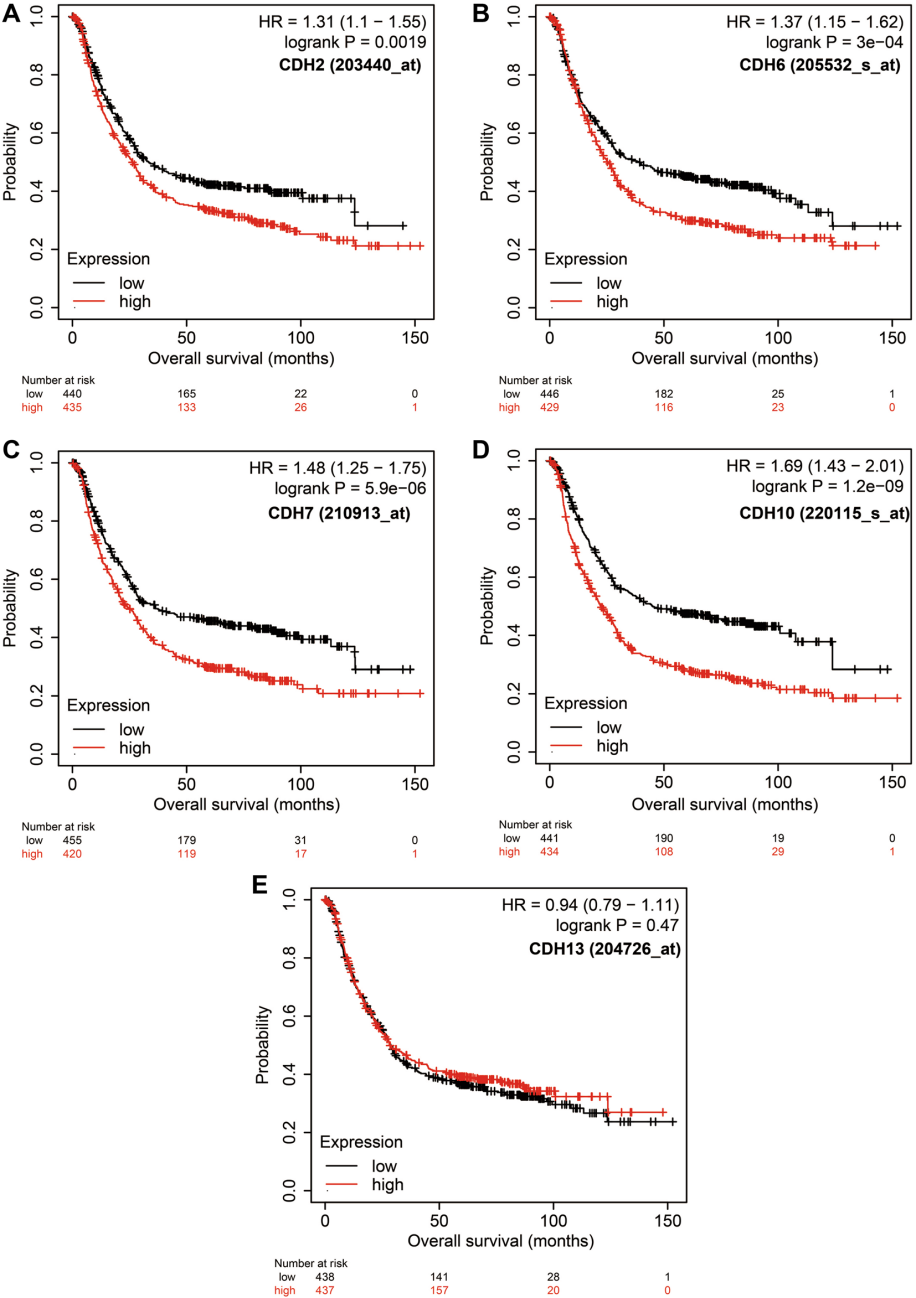
Kaplan–Meier survival curves of prognostic-related CDH genes in Kaplan–Meier plotter cohort. (**A**) Kaplan–Meier curve of CDH2; (**B**) Kaplan–Meier curve of CDH6; (**C**) Kaplan–Meier curve of CDH7; (**D**) Kaplan–Meier curve of CDH10; (**E**) Kaplan–Meier curve of CDH13.

**Figure 8:**
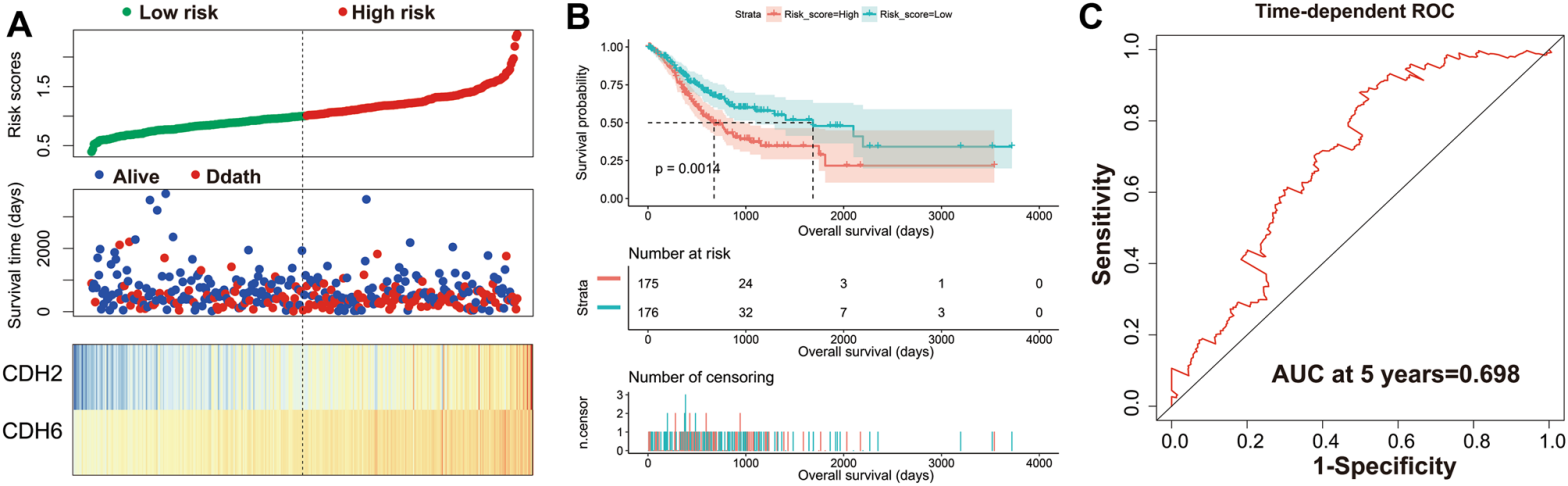
Survival analysis of risk score in GC patients of TCGA cohort. (**A**) Risk Score model and survival time distribution map of GC patients; (**B**) Kaplan–Meier curves of risk score in GC; (**C**) Time-dependent ROC curve of risk score in GC prognosis.

**Figure 9:**
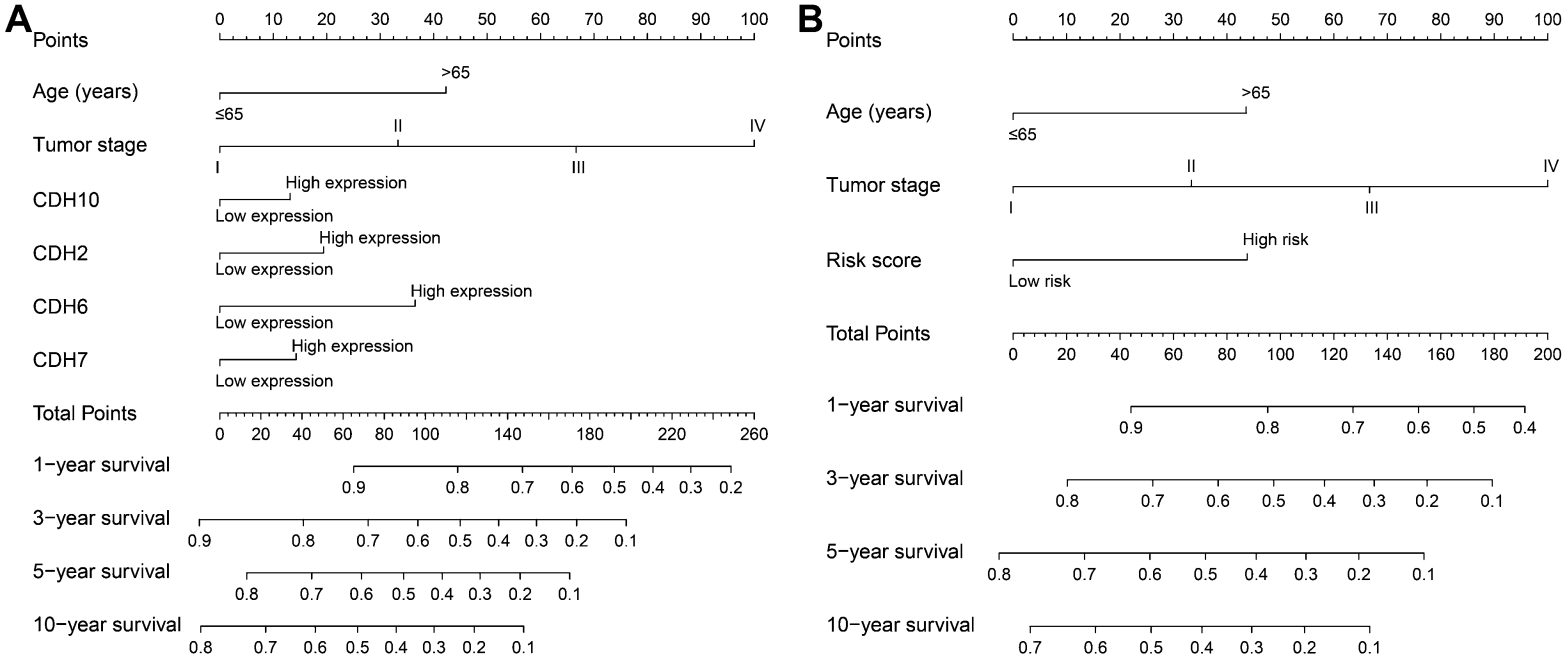
Nomogram of prognostic CHD genes and risk score in TCGA cohort of GC patients. (**A**) Nomogram of prognostic CHD genes in GC patients; (**B**) Nomogram of risk score in GC patients.

A total of 408 GC-derived RNA-seq samples were obtained from TCGA website. These included 32 para-carcinoma and 375 tumor tissue samples. The pheatmap package in R was used to draw a heat map of the CDH gene expression distribution in GC para-carcinoma and tumor tissue samples (Fig. 3). In the CDH gene family, 12 genes were found to be differentially expressed between GC para-carcinoma and tumor tissue. Five of these genes were significantly down-regulated in the cancer tissue, while seven were significantly up-regulated (Fig. 4, Table 1). When adjusting for tumor stage and age in the subsequent multivariate Cox proportional hazard regression model of the CDH genes found that five CDH genes were significantly associated with GC prognosis. These five prognostic genes included CDH6, CDH10, CDH7, CDH2, and CDH13 (Table 1, Fig. 5A–E). High expression of each these five genes was significantly associated with poor GC prognosis. Among these five prognostic CDH genes, with a time-dependent area under the ROC curve of 0.680, CDH6 was found to have the highest accuracy in predicting 5-year survival in GC patients (Fig. 6A–E). To verify the prognostic value of these five CDH genes in GC, GC patient data derived from the Kaplan–Meier plotter database were used as a validation cohort. CDH6, CDH10, CDH7 and CDH2 were found to be significantly correlated with GC prognosis, and that high expression of these genes were significantly correlated with poor clinical outcome in GC cases (*P* < 0.01 for all log-rank values, Fig. 7A–D). No significant correlation was found between CDH13 mRNA expression levels and GC prognosis (log-rank *P* = 0.47, Fig. 7E). An expression matrix of the TCGA cohort was therefore used for the CDH6, CDH10, CDH7 and CDH2 genes to construct the prognostic risk score model. Through step function screening, a prognostic risk score model was constructed based on the expression of CDH2 and CDH6. High- and low-risk GC patients were defined according to the median risk score value. The risk score calculation model was as follows: risk score = (0.0979 × CDH2 expression) + (0.1841 × CDH6 expression). In the analysis of the prognostic risk scores, it was observed that high-risk GC patients were significantly associated with poor prognosis (log-rank *P* < 0.001, adjusted *P* < 0.001, HR = 1.910, 95%CI 1.339–2.724, Fig. 8A,B), and that their median survival time of 675 days was shorter than low-risk patients (1686 days). The construction of the prognostic risk score model based on CDH2 and CDH6 was observed to significantly improve the accuracy when predicting the 5-year survival of GC patients (AUC = 0.698, Fig. 8C). In order to evaluate the contribution of CDH genes to the prognosis of GC patients, CDH prognostic genes and associated risk scores were used to construct the nomogram models. Using the nomogram model, it was found that tumor stage had the greatest contribution to prognosis. Among the CDH prognostic genes, CDH6 had a greater contribution to GC prognosis than the other prognostic CDH genes (Fig. 9A). In the risk-score nomogram model, tumor stage was found to have had a greater contribution to the prognosis of GC than risk score, but risk score had a greater contribution to GC prognosis than single CDH prognostic genes (Fig. 9B).

**Table 1:** Differential expression analysis and survival analysis results of cadherin family genes in patients with GC.

Genes	Differential expression analysis			Survival analysis			
	Log_2_FC	*P* value	FDR	HR	Low 95%CI	High 95%CI	*P* value^a^
CDH6	0.62785	0.001087	0.002528	1.743046	1.244591	2.441131	0.001224
CDH10	− 1.38304	0.000356	0.000924	1.481766	1.063939	2.063682	0.019983
CDH7	2.587741	5.03E−05	0.000154	1.427849	1.025923	1.987238	0.034712
CDH2	− 0.70155	0.018561	0.032195	1.398358	1.003822	1.947959	0.047421
CDH13	0.149213	0.409142	0.483745	1.394074	1.000662	1.942156	0.049544
CDH19	− 2.72179	1.47E−17	2.83E−16	1.386825	0.996992	1.929086	0.052128
CDH11	1.29337	1.69E−08	9.33E−08	1.346713	0.967842	1.873896	0.07739
CDH24	1.099836	9.75E−13	9.98E−12	0.746382	0.536451	1.038466	0.082569
CDH23	− 0.81658	0.000126	0.000359	1.319771	0.948715	1.835954	0.099481
CDH18	− 1.13685	0.004483	0.00904	1.263239	0.908346	1.756789	0.164927
CDH20	− 1.49485	1.41E−09	9.06E−09	1.237657	0.890568	1.72002	0.204163
CDH9	1.352549	0.134758	0.185504	1.233986	0.887168	1.716386	0.211722
CDH17	− 0.07249	0.792939	0.835592	0.854445	0.61482	1.187464	0.348884
CDH5	0.435058	0.008582	0.016185	1.124284	0.809108	1.562232	0.485212
CDH8	− 1.31559	5.62E−07	2.45E−06	1.121027	0.807453	1.556376	0.494966
CDH26	− 0.36218	0.18336	0.242987	1.117019	0.80406	1.551789	0.5094
CDH22	− 0.71153	0.111335	0.156697	0.909061	0.653628	1.264314	0.571061
CDH1	0.242224	0.252366	0.319951	1.08824	0.783617	1.511282	0.613776
CDH16	1.476062	0.007818	0.014887	0.92953	0.668814	1.291879	0.663485
CDH3	1.87272	8.07E−09	4.66E−08	0.951648	0.685421	1.321282	0.767231
CDH4	0.679658	0.025361	0.042489	1.02823	0.740096	1.42854	0.868203
CDH12	2.665141	0.001075	0.002503	1.010717	0.727919	1.403382	0.949247
CDH15	0.64474	0.041405	0.065536	0.995598	0.716718	1.382991	0.979009

^a^Adjusted for age and tumor stage in the multivariate Cox proportional hazard regression model.

### Functional enrichment analysis of cadherin genes in GC

**Figure 10:**
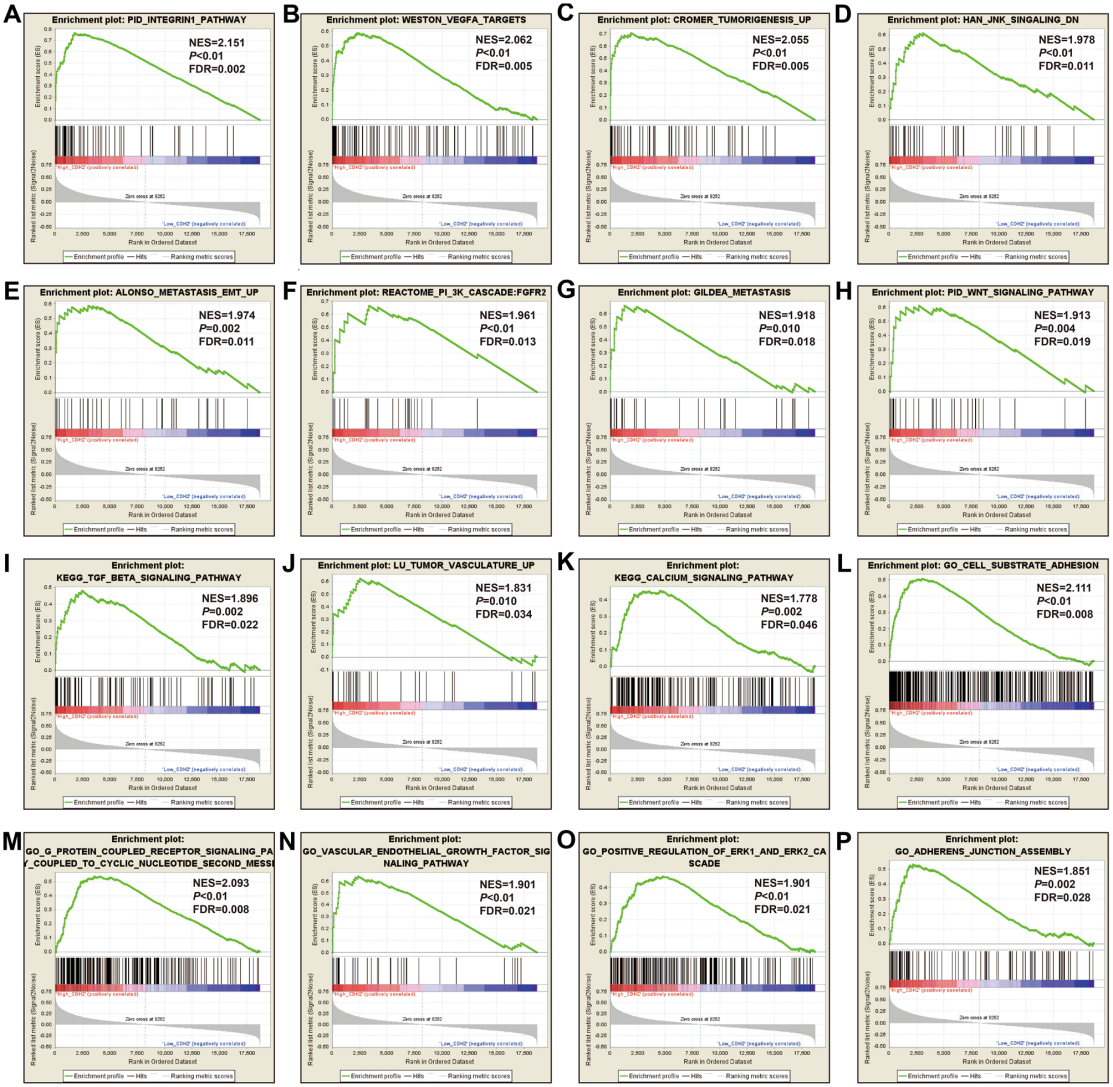
GSEA analysis between low- and high-CDH2 phenotypes in TCGA GC cohort. (**A**) integrin1 pathway; (**B**) VEGFA targets; (**C**) tumor tumorigenesis; (**D**) JNK signaling dn; (**E**) metastasis EMT up; (**F**) PI3K cascade: FGFR2; (**G**) metastasis; (**H**) Wnt signaling pathway; (**I**) TGF beta signaling pathway; (**J**) tumor vasculature up; (**K**) calcium signaling pathway; (**L**) cell substrate adhesion; (**M**) G protein coupled receptor signaling pathway coupled to cyclic nucleotide second messenger; (**N**) vascular endothelial growth factor signaling pathway; (**O**) positive regulation of Erk1 and Erk2 cascade; (**P**) adherens junction assembly.

**Figure 11:**
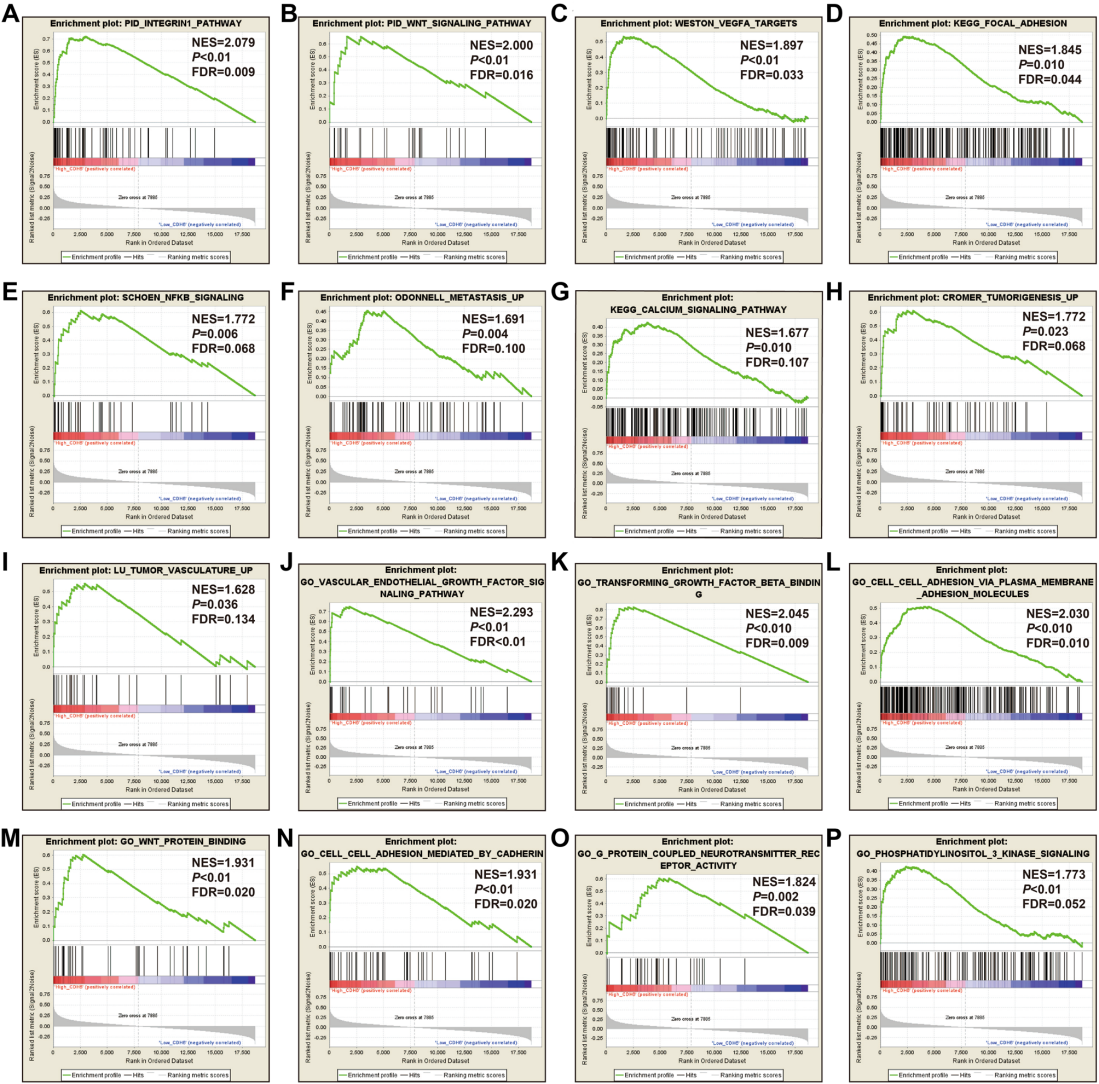
GSEA analysis between low- and high-CDH6 phenotypes in TCGA GC cohort. (**A**) integrin1 pathway; (**B**) Wnt signaling pathway; (**C**) VEGFA targets; (**D**) focal adhesion; (E) NF-κB signaling; (**F**) metastasis up; (**G**) calcium signaling pathway; (**H**) tumorigenesis up; (**I**) tumor vasculature up; (**J**) vascular endothelial growth factor signaling pathway; (**K**) transforming growth factor beta binding; (**L**) cell cell adhesion via plasma membrane adhesion molecules; (**M**) Wnt protein binding; (**N**) cell cell adhesion mediated by cadherin; (**O**) G protein coupled neurotransmitter receptor activity; (**P**) phosphatidylinositol 3 kinase signaling.

**Figure 12:**
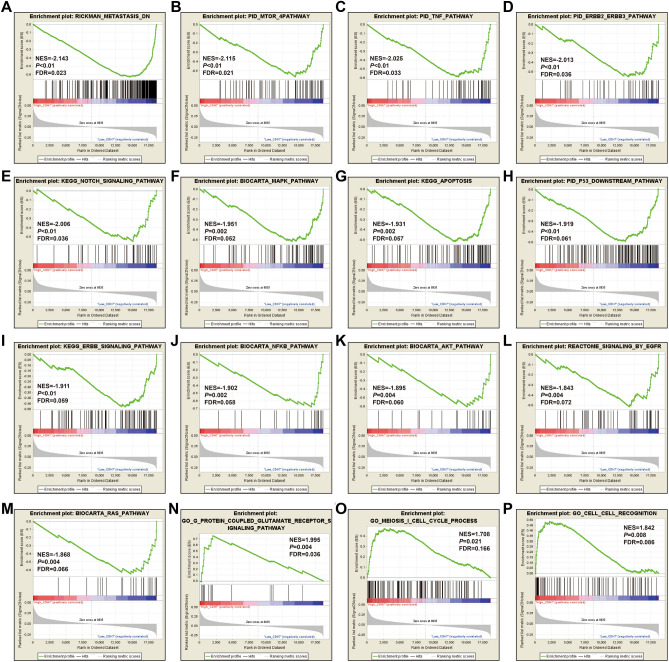
GSEA analysis between low- and high-CDH7 phenotypes in TCGA GC cohort. (**A**) metastasis dn; (**B**) mTOR 4 pathway; (**C**) TNF pathway; (**D**) ERBB2/ERBB3 pathway; (**E**) Notch signaling pathway; (**F**) MAPK pathway; (**G**) apoptosis; (**H**) p53 downstream pathway; (**I**) ERBB signaling pathway; (**J**) NF-κB pathway; (**K**) Akt pathway; (**L**) signaling by EGFR; (**M**) Ras pathway; (**N**) G protein coupled glutamate receptor signaling pathway; (**O**) cell cycle process; (**P**) cell–cell recognition.

**Figure 13:**
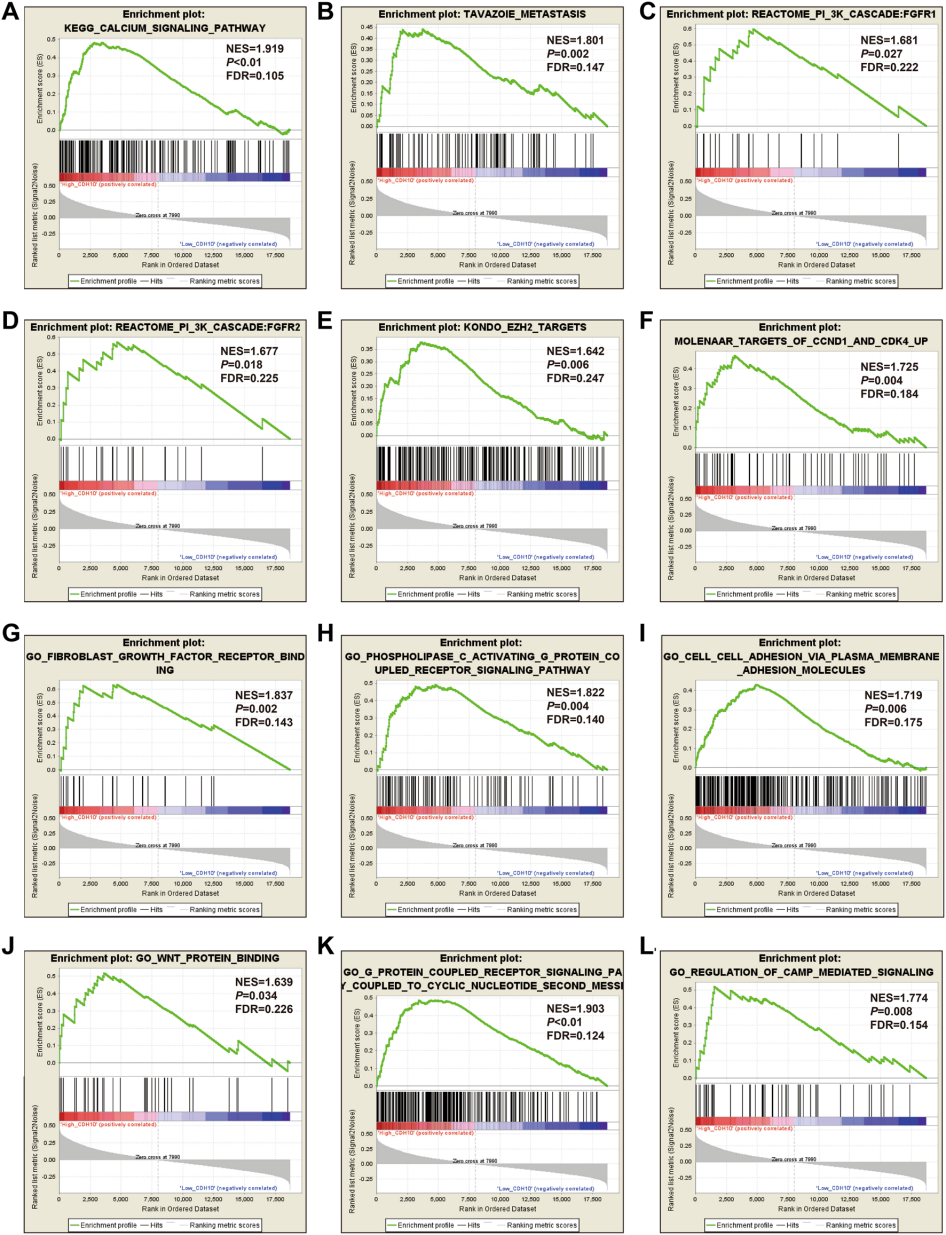
GSEA analysis between low- and high-CDH10 phenotypes in TCGA GC cohort. (**A**) calcium signaling pathway; (**B**) metastasis; (**C**) PI3K cascade: FGFR1; (**D**) PI3K cascade: FGFR2; (**E**) EZH2 targets; (**F**) targets of CCND1 and CDK4 up; (**G**) fibroblast growth factor receptor binding; (**H**) phospholipase C activating G protein coupled receptor signaling pathway; (**I**) cell cell adhesion via plasma membrane adhesion molecules; (**J**) Wnt protein binding; (**K**) G protein coupled receptor signaling pathway coupled to cyclic nucleotide second messenger; (**L**) regulation of cAMP mediated signaling.

**Figure 14:**
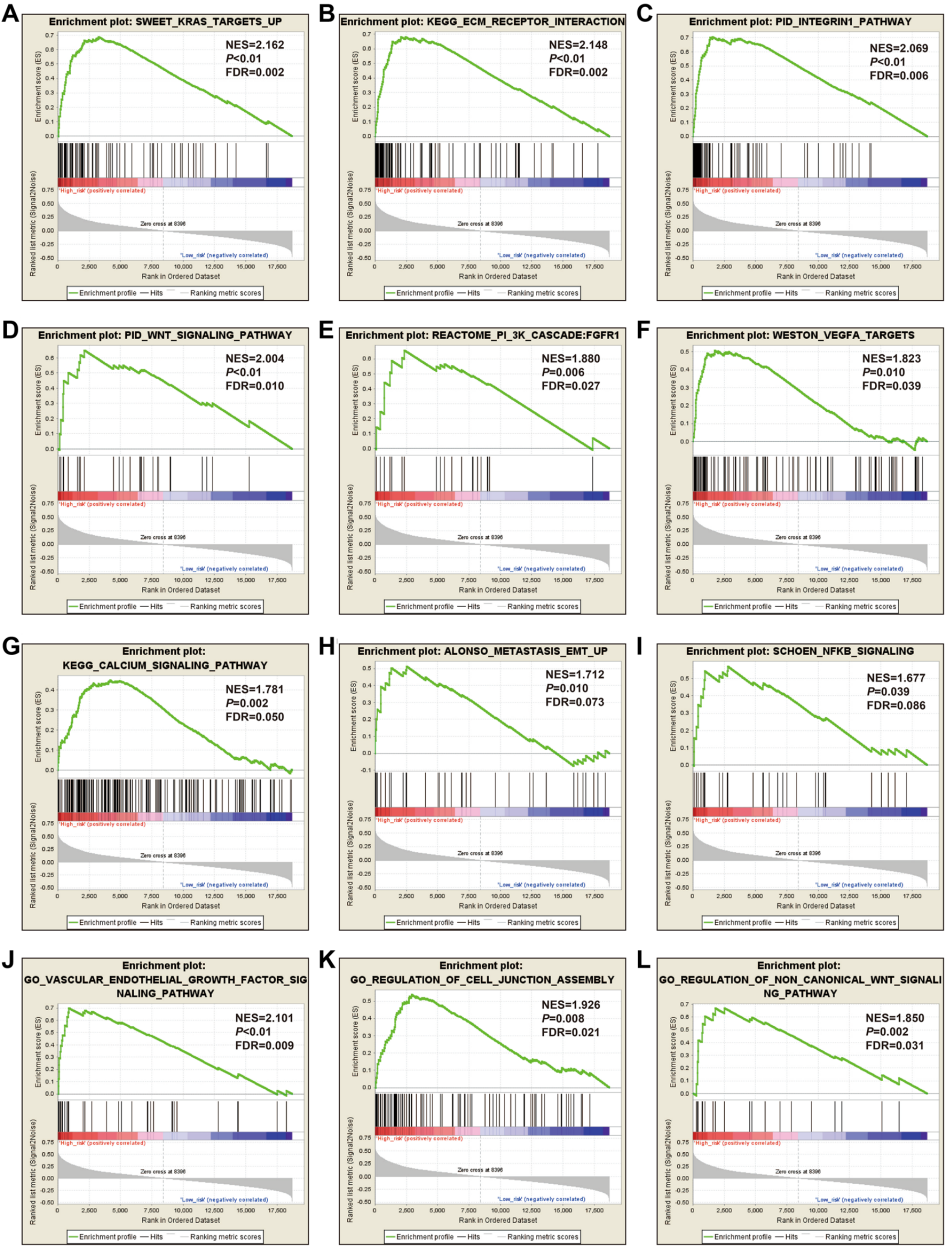
GSEA analysis between low- and high-risk phenotypes in TCGA GC cohort. (**A**) Kras targets up; (**B**) ECM receptor interaction; (**C**) integrin1 pathway; (**D**) Wnt signaling pathway; (**E**) PI3K cascade: FGFR1; (**F**) VEGFA targets; (**G**) calcium signaling pathway; (**H**) metastasis EMT up; (**I**) NF-κB signaling; (**J**) vascular endothelial growth factor signaling pathway; (**K**) regulation of cell junction assembly; (**L**) regulation of non canonical Wnt signaling pathway.

In order to understand the potential biological mechanisms involved in the prognostic CHD genes and risk scores in GC, GSEA was used to perform enrichment analysis for different CDH expression levels or risk score phenotypes. GSEA of CDH2 in TCGA cohort found that a high CDH2 expression phenotype was significantly involved in several systems and pathways. These included the integrin1 pathway, VEGFA targets, tumor tumorigenesis, JNK signaling dn, metastasis epithelial-mesenchymal transition (EMT) up, PI3K cascade: FGFR2, metastasis, Wnt signaling pathway, TGF beta signaling pathway, tumor vasculature up, calcium signaling pathway, cell substrate adhesion, G protein coupled receptor signaling pathway coupled to cyclic nucleotide second messenger, vascular endothelial growth factor signaling pathway, positive regulation of Erk1 and Erk2 cascade and adherens junction assembly (Fig. 10A–P, Table S2). For CDH6, we found that the high CDH6 expression phenotype was significantly associated with the integrin1 pathway, and Wnt signaling pathway, VEGFA targets, focal adhesion, NF-κB signaling, metastasis up, calcium signaling pathway, tumorigenesis up, tumor vasculature up, vascular endothelial growth factor signaling pathway, transforming growth factor beta binding, cell cell adhesion via plasma membrane adhesion molecules, Wnt protein binding, cell cell adhesion mediated by cadherin, G protein coupled neurotransmitter receptor activity and phosphatidylinositol 3 kinase signaling (Fig. 11A–P, Table S3). For CDH7, the differences between low- and high-CDH7 expression phenotypes were significant in metastasis dn, mTOR 4 pathway, TNF pathway, ERBB2/ERBB3 pathway, Notch signaling pathway, MAPK pathway, apoptosis, p53 downstream pathway, ERBB signaling pathway, NF-κB pathway, Akt pathway, signaling by EGFR, Ras pathway, G protein coupled glutamate receptor signaling pathway, cell cycle process and cell–cell recognition (Fig. 12A–P, Table S4). The high CDH10 expression phenotype was significantly involved in calcium signaling pathway, metastasis, PI3K cascade: FGFR1, PI3K cascade: FGFR2, EZH2 targets, targets of CCND1 and CDK4 up, fibroblast growth factor receptor binding, phospholipase C activating G protein coupled receptor signaling pathway, cell cell adhesion via plasma membrane adhesion molecules, Wnt protein binding, G protein coupled receptor signaling pathway coupled to cyclic nucleotide second messenger and regulation of cAMP mediated signaling (Fig. 13A–L, Table S5). The high-risk score phenotype was significantly involved in Kras targets up, ECM receptor interaction, integrin1 pathway, Wnt signaling pathway, PI3K cascade: FGFR1, VEGFA targets, calcium signaling pathway, metastasis EMT up, NF-κB signaling, vascular endothelial growth factor signaling pathway, regulation of cell junction assembly and regulation of non canonical Wnt signaling pathway (Fig. 14A–L, Table S6).

### Drug screening for GC risk score model

**Figure 15:**
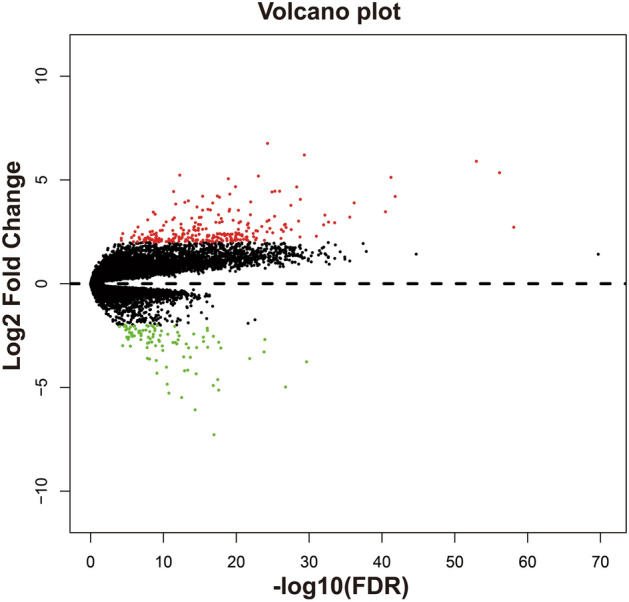
Volcano plot of DEGs between low- and high-risk phenotypes in TCGA GC cohort.

**Figure 16:**
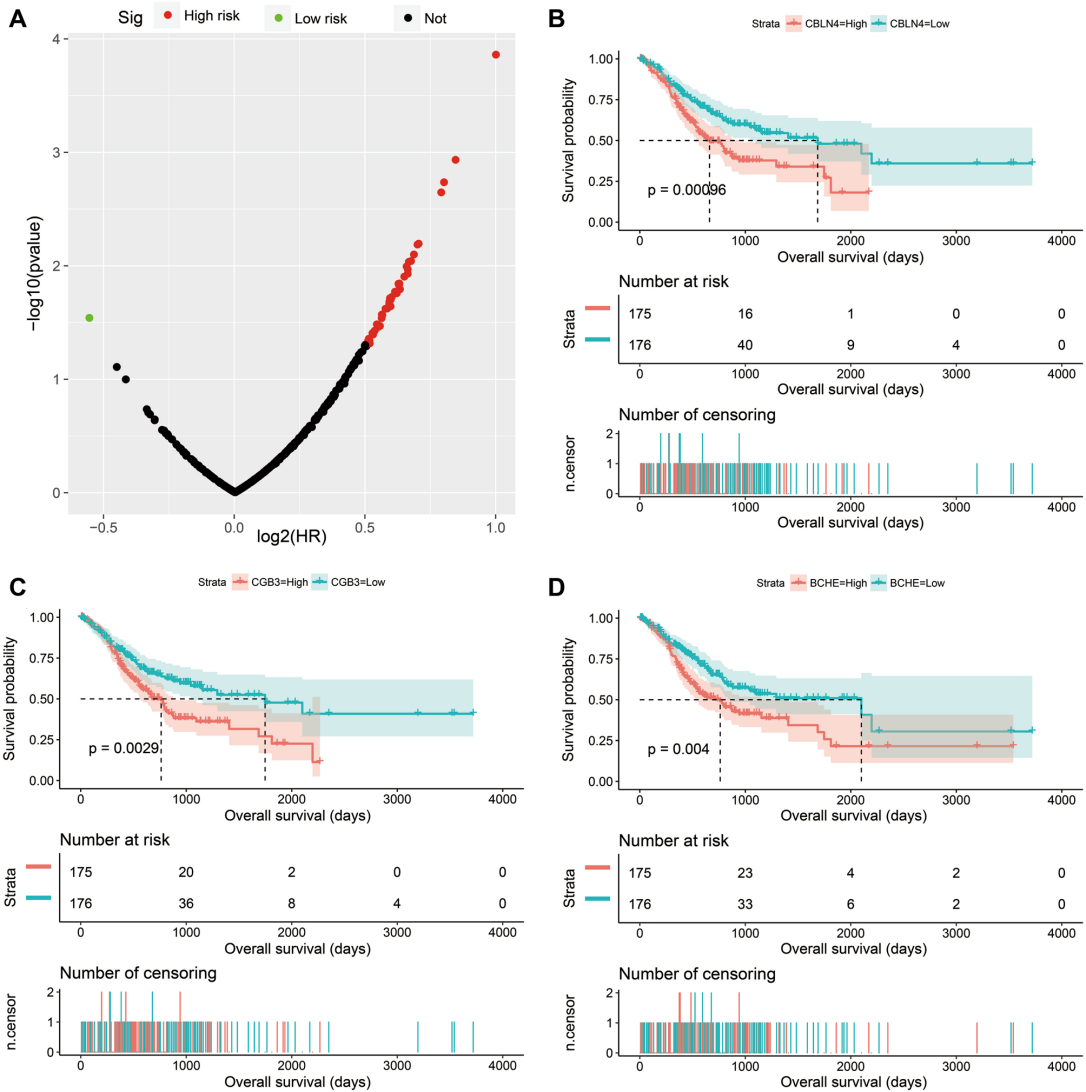
Survival analysis results of DEGs between low- and high-risk phenotypes in TCGA cohort. (**A**) Volcano plot of DEGs survival analysis results; (**B**) Kaplan–Meier curves of CBLN4 in GC; (**C**) Kaplan–Meier curves of CGB3 in GC; (**D**) Kaplan–Meier curves of BCHE in GC.

**Figure 17:**
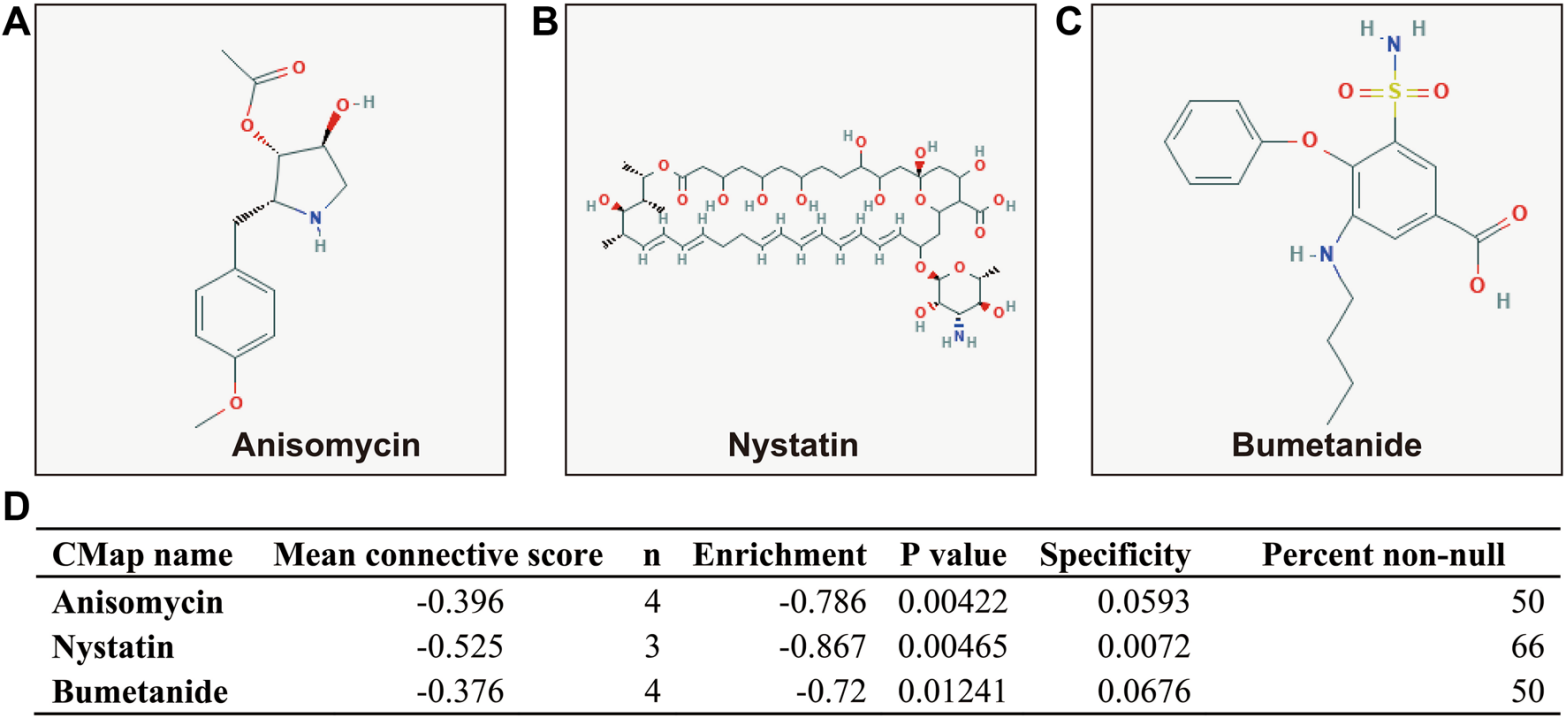
CMAP analysis results of low- and high-risk phenotypes in TCGA cohort. (**A**) Chemical structure of anisomycin; (**B**) Chemical structure of nystatin; (**C**) Chemical structure of bumetanide; (**D**) CMap analysis results list.

**Figure 18:**
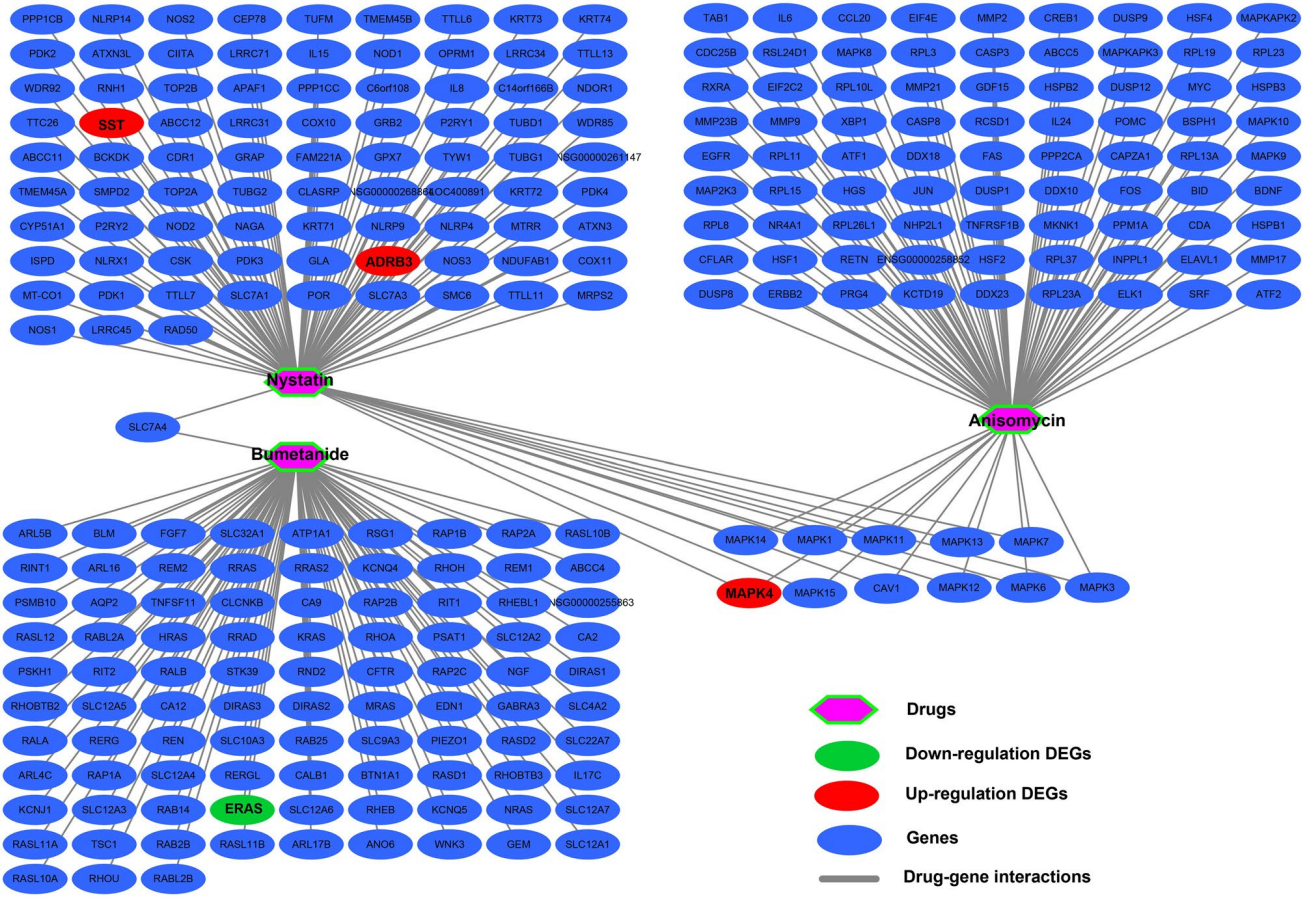
Drug–gene interaction networks of the three targeted drugs for different risk phenotypes in GC.

In order to screen targeted therapeutic drugs for GC risk scores, *edgeR* was used. This enabled the screening of DEGs between high- and low-risk phenotypes. A total of 344 DEGs were obtained across high- and low-risk phenotypes (Fig. 15, Table S7). The heat map for these DEGs is shown in Fig. S1. A multivariate Cox proportional hazards regression model, corrected for tumor stage and age, was then used for prognostic analysis. A total of 45 DEGs were observed to be significantly correlated with GC prognosis in the TCGA cohort (Table S8, Fig. 16A). The three most significant DEGs included cerebellin 4 precursor (CBLN4, log-rank *P* = 0.00096, Fig. 16B), chorionic gonadotropin subunit beta 3 (CGB3, log-rank *P* = 0.0029, Fig. 16C) and butyrylcholinesterase (BCHE, log-rank *P* = 0.004, Fig. 16D). Through functional enrichment analysis of the DEGs, it was found that these DEGs may participate in calcium signaling pathway, ECM-receptor interaction, cAMP signaling pathway, cGMP-PKG signaling pathway, gastric acid secretion, adenylate cyclase-activating G-protein coupled receptor signaling pathway, calcium-dependent protein binding and G-protein coupled receptor signaling pathway, coupled to cyclic nucleotide second messenger function, was also found to be impacted. These biological processes and signaling pathways may be the potential mechanisms driving the differences in clinical outcome among GC patients across high- and low-risk phenotypes (Table S9). The DEGs were used to conduct targeted drug screening through the CMap online tool. Three potential small molecule compounds, anisomycin, nystatin and bumetanide, were found to be linked to the GC risk score. The chemical structures of the three drugs are shown in Fig. 17A–C, while the CMap analysis results are summarized in Fig. 17D. The STITCH online tool was used to construct the drug–gene interaction network. Bumetanide was found to potentially play a role in GC by targeting *ERAS* (ES cell expressed Ras) genes, while nystatin may play a role in GC by targeting SST (somatostatin) and ADRB3 (adrenoceptor Beta 3) genes (Fig. 18).

### Relationship between prognostic CDH genes expression and tumor immune infiltration abundance

**Figure 19:**
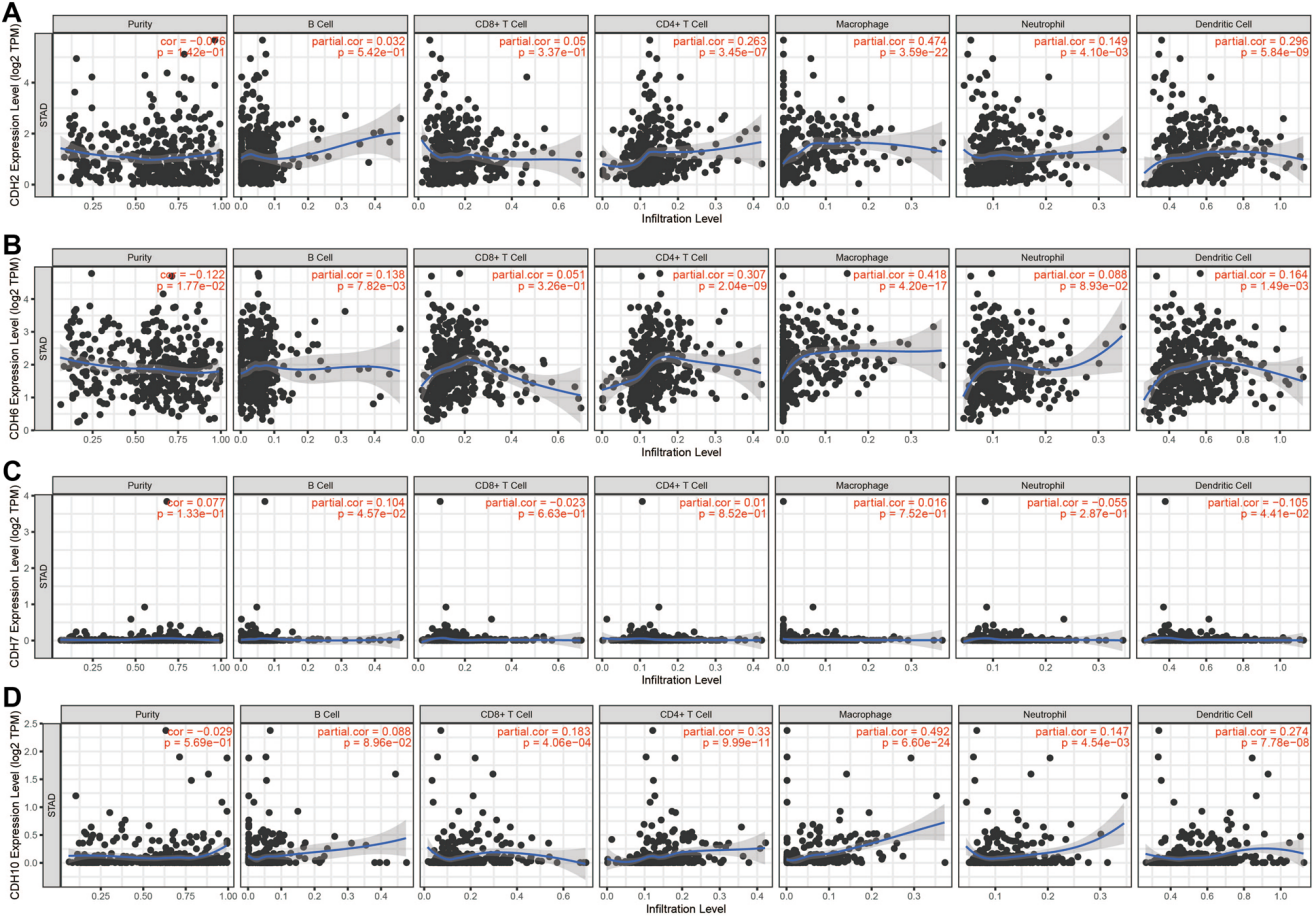
Relationship between prognostic CDH genes expression and tumor immune infiltration abundance in GC tumor tissues**.** (**A**) Relationship between CDH2 expression and tumor immune infiltration abundance; (**B**) Relationship between CDH6 expression and tumor immune infiltration abundance; (**C**) Relationship between CDH7 expression and tumor immune infiltration abundance; (**D**) Relationship between CDH10 expression and tumor immune infiltration abundance.

By analyzing the relationship between the prognostic CDH genes and the abundance of tumor immune infiltration (Fig. 19A–D), we found that expression level of CDH6 (r = 0.138, *P* = 7.82 × 10^−3^) and CDH7 (r = 0.104, *P* = 4.57 × 10^−2^) were significantly related to B cell infiltration in GC tumor tissues. For CD8 + T cell, we found that the expression level of CDH10 (r = 0.183, *P* = 4.06 × 10^−4^) in GC tumor tissues were closely related to CD8 + T cell immune infiltration. The expression level of CDH2(r = 0.263, *P* = 3.54 × 10^−7^), CDH6 (r = 0.307, *P* = 2.04 × 10^−9^) and CDH10 (r = 0.33, *P* = 9.99 × 10^−11^) in GC tumor tissues were closely related to CD4 + T cell immune infiltration. Tumor immune infiltration abundance of macrophage also shown a significantly associated with CDH2 (r = 0.474, *P* = 3.59 × 10^−22^), CDH6 (r = 0.418, *P* = 4.20 × 10^−17^) and CDH10 (r = 0.492, *P* = 6.60 × 10^−24^) in GC tumor tissues. Tumor immune infiltration abundance of neutrophil were closely correlated with CDH2 (r = 0.149, *P* = 4.10 × 10^−3^) and CDH10 (r = 0.147, *P* = 4.54 × 10^−3^) expression. All these four prognostic genes were significant associated with dendritic cell Tumor immune infiltration abundance: CDH2 (r = 0.296, *P* = 5.84 × 10^−9^), CDH6 (r = 0.164, *P* = 1.49 × 10^−3^), CDH6 (r = − 0.105, *P* = 4.41 × 10^−2^) and CDH10 (r = 0.274, *P* = 7.78 × 10^−8^).

## Discussion

In previous studies, the CDH gene family have reportedly played an important role in cancers, especially in its prognosis^6–8^. Yu et al. found that the rs643555C > T site of CDH2 can be used as a prognostic marker for prostate cancer (PCa)^9^, while Gao et al. showed that CDH2 is the target for miR-194 using luciferase reporter gene analysis. In vitro cell experiments confirmed that miR-194 can directly target CDH2 to regulate PCa cell survival and inhibit tumor growth in vivo^10^. Zhang et al. found that circ_000926 exerts a tumor suppressive effect through the circ_000926-miRNA-411-CDH2 competing endogenous RNA (ceRNA) regulatory network. Thus, when the expression of CDH2 in renal carcinoma increased, the anti-tumor effect of CDH2 in renal cell carcinoma decreased^11^. Shi et al. similarly found that lncAPP can enhance PCa cell proliferation and promote cell migration and invasion through the ceRNA regulatory network via lncapP-miR218-ZEB2/CDH2 interaction^12^. Chen et al. found that CDH2 is closely related to glioma tumor grade after analyzing data from a glioma cohort constructed through multiple public data banks. The prognostic analysis shows that high CDH2 expression is closely related to poor glioma prognoses and that there is no subsequent benefit obtained from temozolomide treatment^13^. Zhou et al. found that CDH2 can significantly promote angiogenesis in lung adenocarcinoma (LUAD) and participate in regulating the sensitivity of angiogenesis antagonists^14^. Downstream analysis suggests that CDH2 may play a role in regulating MAPK/ERK and MAPK/JNK signaling pathways in LUAD^14^. LUAD patients with high CDH2 expression in tumor-derived endothelial cells are significantly associated with an unfavorable prognosis, tumor stage and visceral pleural metastasis^14^. Qiu et al. found that CDH2 is significantly up-regulated in tumor tissue and serves as a hub gene in the pathogenesis of papillary thyroid cancer (PTC)^15^. In gallbladder cancer, CDH2 is not only closely related to a poor prognosis, but also to clinicopathological features such as tumor size, invasion, and lymph node metastasis^16^. Zhang et al. found that the potential mechanism of the actin-related protein 2/3 complex subunit 2 as an oncogene in GC is through the downstream regulation of CDH2 and other genes^17^. Abnormal *CDH2* expression is due to the widespread occurrence of EMT in diffuse-type GC when compared to intestinal-type GC^18^. Gao et al. found that miR-145 was significantly down-regulated in GC and metastatic tumor tissue. Results of functional experiments suggest that miR-145 can inhibit the migration, invasion and metastatic ability of GC cells. Analysis through a luciferase reporter system suggested that CDH2 was the target gene for miR-145, and that miR-145 can reverse its ability to inhibit the migration, invasion and metastasis of GC cells through targeted regulation of CDH2^19^. Chang et al. found that miR‑205 has a tumor suppressing effect and inhibits EMT in GC. Upon in vitro transfection of GC cell lines with miR‑205, CDH2 expression was down-regulated and was therefore considered to be a mesenchymal marker^20^.

Sun et al. have developed a 12-gene signature, which includes CDH6, that can predict the sensitivity and resistance of glioma patients to targeted molecular therapy, while additionally serving as an independent prognostic factor for glioma patients^21^. By comparing the serum of patients with high-grade serous carcinoma against control samples, Graumann et al. found that CDH6 was significantly elevated in patients with high-grade serous carcinoma^22^. Xu et al. found that CDH6 is negatively correlated with PCa tumor stage and prostate-specific membrane antigen levels. It was therefore suggested that CDH6 may be involved in the regulation of PSMA-related prostate cancer metastasis mechanisms^23^. While Goeppert et al. found that CDH6 serves as a tumor suppressor gene in cholangiocarcinoma in that its expression is significantly lower in cholangiocarcinoma tumor tissue and that the prognosis of cholangiocarcinoma patients with low expression of CDH6 is poor^24^, Zuo et al. found that it is significantly up-regulated in nasopharyngeal carcinoma (NPC) tumor tissue and can promote NPC metastasis by inducing EMT^25^. Gugnoni et al. found that CDH6 in papillary thyroid cancers (PTC) can be used as a pro-metastatic gene and serves as a biomarker in PTC cases with high invasive ability^26^. There are additional studies suggesting that CDH6 is an important EMT marker in PTC as it can promote PTC metastasis by participating in the regulation of autophagy, thus leading to a poor prognosis^27^. Ma et al. suggest that CDH6 is significantly up-regulated in patients with lymph node metastasis in oral squamous cell carcinoma (OSCC) and that patients have a poor prognosis^28^. It was further suggested that, when combined with other adhesion factor-related genes, CDH6 can be used as an important biomarker for OSCC lymph node metastasis and prognosis^28^. An in vivo study by Karthikeyan et al. confirmed that CDH6 was inhibited by mutant p53 in high-grade serous ovarian cancer, which suggests that CDH6 may be a biomarker for this condition^29^. While Ji et al. observed that CDH6 is highly expressed in osteosarcoma, prognostic analysis found that it is closely related to a poor prognosis in such cases. Functional experiments suggest that miRNA can significantly inhibit the proliferation, invasion and migration of osteosarcoma cell lines through targeted regulation of CDH6. This study shows that CDH6 can be used as an important metastasis and prognostic biomarker of osteosarcoma^30^. Through next-generation sequencing, Liu et al. observed that CDH7 is a new pulmonary sarcomatoid carcinoma mutation^31^, while Cobrinik et al. found that CDH7 is related to the central nervous system metastasis process through SNP microarray analysis of stage 4 neuroblastomas^32^. CDH7 has also been reported to participate in the negative regulation of melanoma cell migration in malignant melanomas^33^. CDH10 is a specific adhesion molecule of the blood–brain barrier, and plays a key role in the development and maintenance of this system^34^. A study using a TCGA dataset cohort observed that low expression of CDH10 is closely related to poor prognosis in breast cancer patients^35^, while Jinawath et al. found that CDH10 is closely related to the occurrence of familial pancreatic cancer (FPC) after conducted chromosome studies and sequencing analyses on FPC samples^36^. A study by An et al. found that unconventional frameshift mutations in CDH10 can lead to inactivation of cell adhesion-related functions in GC and colorectal cancers (CRC); a potential characteristic feature associated with high-frequency microsatellite instability in such cases^37^. Jiang et al. analyzed 630 patients with stage I endometrioid-type endometrial cancer (EEC) and found that CDH10 gene mutations can promote the initiation the lung metastasis of EEC^38^. Yu et al. found the first CDH10 mutation in CRC patients, and indicate that the prognostic signature of this mutation was good when combined with four other gene mutations in CRC patients^39^. Li et al. performed whole exome sequencing on patients with lung squamous cell carcinoma (SQCC) and found that CDH10 is a high-frequency, SQCC-associated mutation^40^. In vitro experiments confirmed that CDH10 plays the role of a tumor suppressor gene in SQCC^40^. Collectively, CDH genes have been widely reported in a variety of cancers, with most of the studies investigating the biological role of these genes in cancer cell adhesion and their associated prognostic value.

For functional analysis, GSEA results suggest that the functional mechanisms of the four prognostic CDH genes and their associated risk scores may be involved in multiple, classic, cancer-related signaling pathways in GC. These include the Wnt and PI3K signaling pathways^41–43^. For the drugs screened in this study, while anisomycin and bumetanide were found to have been reported for cancer therapy in previous studies, studies concerning the anti-tumor effect of nystatin were absent. In vivo experiments showed that bumetanide has anti-angiogenic effects in CRC and that this drug may have clinical application value in CRC patients^44^. Similarly, photodynamic therapy combined with bumetanide was, through in vivo experiments, shown to significantly inhibit the growth of rat gliomas, reduce the peritumoral edema caused by simple photodynamic therapy, and thus improve the survival of rats^45^. Bumetanide can also enhance cisplatin-induced apoptosis of mesothelioma cells, thereby augmenting the sensitivity of chemotherapy drugs^46^. Anisomycin can have a direct killing effect in hepatocellular carcinoma and has an anti-tumor effect mediated by natural killer cell immunotherapy^47^. While anisomycin inhibits angiogenesis, proliferation and invasion in ovarian cancer cells by regulating the lncRNA-Meg3/miR-421/PDGFRA-Notch pathway axis^48^, a separate study found that lncRNA BACE1-AS is a new anisemycin target in ovarian cancer^49^. Anisomycin can also inhibit the proliferation of CRC cells and can also enhance the anti-tumor effect of 5-fluorouracil^50^. Similarly, anisomycin has an anti-tumor effect in osteosarcoma that can inhibit osteosarcoma cell line proliferation by blocking the cell cycle, inducing apoptosis by caspase-dependent inducement, and enhancing the patient’s sensitivity to doxorubicin^51^. In summary, anisomycin has been reported to have anti-tumor effects in a variety of cancers, and can increase the sensitivity of chemotherapy drugs. However, the anti-tumor effect of anisomycin in gastric cancer has never been reported. Through bioinformatics, this study is the first to predict that this anti-tumor drug can act on gastric cancer. Regarding the research on tumor immune infiltration, we have not found any reports about CDH and tumor immune infiltration of GC in the previous studies. The present study is the first report to investigate the relationship between prognostic CDH genes mRNA expression level and GC tumor immune infiltration.

This study still has some limitations that need to be explained. First, in the Kaplan–Meier plotter database, patients in this cohort were derived from multiple Gene Expression Omnibus datasets. It was therefore challenging to use the same method to verify the target drugs found in the TCGA cohort against the verification cohort. Second, in vivo and in vitro experiments to verify the functional mechanism of the CDH family genes in relation to the drugs screened in this study are lacking. Third, since gastric cancer data from the Kaplan–Meier plotter cohort could not obtain integrated expression values, we could not use the Kaplan–Meier plotter cohort to verify the risk score model. Despite the above shortcomings, this is the first study to conduct a comprehensive analysis into the clinical significance, tumor immune infiltration and molecular mechanism of the CDH genes in relation to GC. Molecular mechanisms screened using the whole genome dataset is thus able to provide guidance and a theoretical basis for future CDH gene studies. Notably, multiple CDH genes that may be used as prognostic markers for GC were found, while three potential GC-targeted drugs were identified. Should the results of this study be verified in a large, multi-center cohort, it will be possible to change the treatment strategy of GC.

## Conclusions

In the present study, CDH2, CDH6, CDH7 and CDH10 were identified and verified as being significantly associated with poor GC prognosis. A risk score signature which can significantly improve the accuracy of predicting the 5-year survival rate of GC patients was constructed based on CDH2 and CDH6. In addition, results from GSEA suggested that the functional mechanisms of the four prognostic CDH genes and their associated risk score may be involved in multiple, classic cancer-related signaling pathways in GC, including the Wnt and PI3K signaling pathways. Lastly, CMap screening identified three small molecule compounds (anisomycin, nystatin and bumetanide) that could be target drugs for risk score adjustment in GC. This study also revealed the relationship between prognostic CDH genes and GC tumor immune infiltration. Since this study is an in silico investigation, our results still need to be verified in future studies using in vivo and in vitro experiments.

## Materials and methods

### Functional enrichment of cadherin genes

Several online bioinformatics analysis tools were used to comprehensively analyze the functions and gene–gene regulatory networks of the CDH genes. While Database was used for Annotation, Visualization, and Integrated Discovery v6.8 (DAVID v6.8, https://david.ncifcrf.gov/home.jsp) was used for functional enrichment analysis of the CDH genes^52^. Gene–gene regulatory networks were evaluated using STRING (https://string-db.org)^53–55^ and GENEMANIA (http://genemania.org/)^56,57^. Using the Pearson correlation coefficient, TCGA GC tumor tissue data was used for CDH-based gene–gene interaction correlation analysis.

### Data acquisition

The test GC dataset was obtained from the TCGA GC cohort (https://portal.gdc.cancer.gov/), while the Kaplan–Meier plotter GC dataset (http://kmplot.com/analysis/index.php?p=service&cancer=gastric) was used as the verification cohort^3,58^. A total of 407 RNA-seq datasets from 380 patients represented in the TCGA GC cohort were included in this study. This included 32 samples from para-carcinoma tissue. TCGA sequencing data was normalized using the *edgeR* package in R^59^. Following screening, RNA-seq data from 351 gastric cancer patients were included with complete clinical parameters taken into account with the subsequent survival analyses^60^. Kaplan–Meier plotter data from a total of 875 GC patients was used in the survival analysis validation cohort. All data in this study were obtained from public databases, and the authors were not involved in any animal or human experiments. Therefore, no additional ethical approval was required for this study.

### Clinical significance of cadherin genes

The *edgeR* package was used to evaluate the differences in the distribution of CDH gene expression in GC tumor and para-carcinoma tissue. The TCGA test and Kaplan–Meier plotter verification cohorts were used to analyze the prognosis of CDH genes in GC patients. In addition, the *survivalROC* package was used to assess the accuracy of GC prognosis when using CDH genes as predictive markers. The *step* function was simultaneously implemented to screen the prognostic CDH genes so as to construct a prognostic signature with higher predictive accuracy. Lastly, the prognostic CDH gene and risk score model was combined with clinical parameters in order to construct two nomograms for individual prognostic GC patient scores.

### Functional enrichment analysis of cadherin genes in GC

In order to further understand the prognostic differences, biological function and mechanism among gastric cancer patients with different CDH gene expression levels, gene set enrichment analysis (GSEA, http://software.broadinstitute.org/gsea/index.jsp) was used^61,62^. Functional differences across different risk score phenotypes were also analyzed using GSEA. GSEA results meeting the following criteria were considered to indicate significant differences between the two phenotypes: |normalized enrichment score (NES)|> 1, nominal *P* < 0.05, and a false discovery rate (FDR) < 0.25. Subsequently, and in order to discover potential therapeutic drugs for GC, the TCGA whole genome RNA sequencing dataset was used to screen differentially expressed genes (DEGs) between different risk score phenotypes, while the connectivity map online database (CMap, https://portals.broadinstitute.org/cmap/) was used for drug discovery. PubChem (https://pubchem.ncbi.nlm.nih.gov) and STITCH (http://stitch.embl.de/) were used to explore the drug chemical structure and gene–drug interaction network respectively. This was done so as to further understand the mechanisms leading to prognostic differences between various risk score phenotypes. The relationship between prognostic CDH genes expression and tumor immune infiltration abundance were carried out by Tumor IMmune Estimation Resource (TIMER: https://cistrome.shinyapps.io/timer/)^63^.

### Statistical analysis

Survival analysis was assessed using Kaplan–Meier curves and Cox proportional hazard regression models. Statistical analysis was performed using SPSS version 22.0 and R version 3.6.2. *P* < 0.05 was considered to indicate statistical significance.

### Ethics approval and consent to participate

Since all datasets of gastric cancer included in the present study were downloaded from open access public database, and the authors were not involved in any animal or human experiments. Therefore, additional approval by an Ethics Committee was not needed.

## Supplementary Information

Supplementary Figure S1.

Supplementary Tables.

## Data Availability

The datasets used during the present study are available from the corresponding author upon reasonable request. All raw data of gastric cancer, which were included in the current study, can be downloaded from TCGA (https://portal.gdc.cancer.gov/) and Kaplan–Meier Plotter (http://kmplot.com/analysis/index.php?p=service&cancer=gastric) .

## Abbreviations

CDH: Cadherin
GC: Gastric cancer
TCGA: The cancer genome atlas
DAVID: Database was used for annotation, visualization, and integrated discovery
GSEA: Gene set enrichment analysis
NES: Normalized enrichment score
FDR: False discovery rate
DEGs: Differentially expressed genes
TIMER: Tumor immune estimation resource
CMap: Connectivity map
