# Supplementary material for: Identification and verification of the molecular mechanisms and prognostic values of the cadherin gene family in gastric cancer
Source: Sci Rep. 2021 Dec 8;11:23674. doi: 10.1038/s41598-021-03086-1 (PMC8655011; doi:10.1038/s41598-021-03086-1)
Supplement: Supplementary file 1 — Supplementary Figure S1. [file 41598_2021_3086_MOESM1_ESM.pdf]

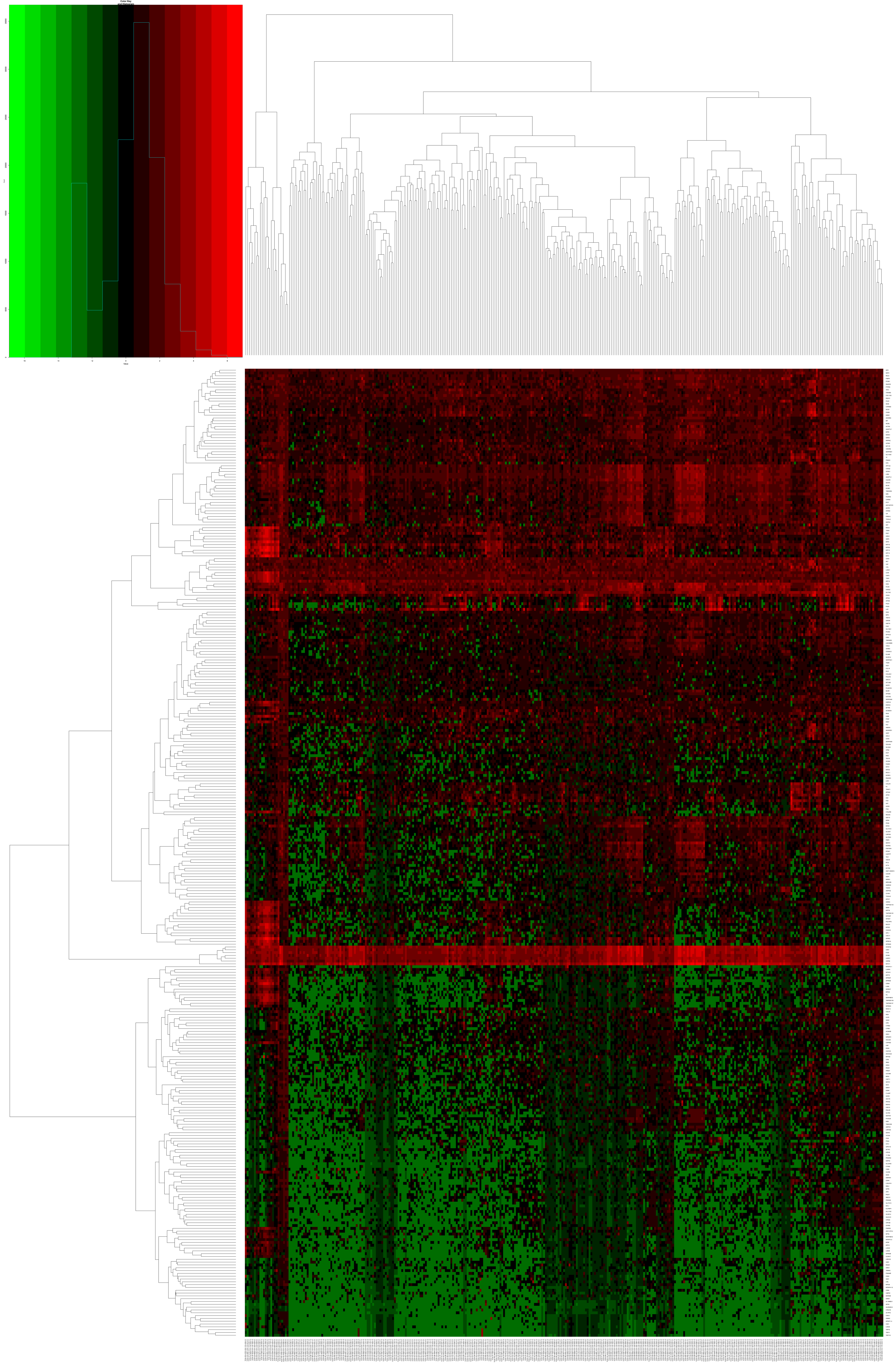

Figure S1.Heat map of DEGs between low- and high-risk score phenotypes.
